# Methods of Calculating Ionization Energies of Multielectron (Five or More) Isoelectronic Atomic Ions

**DOI:** 10.1155/2013/157412

**Published:** 2013-05-16

**Authors:** Peter F. Lang, Barry C. Smith

**Affiliations:** Birkbeck College, University of London, School of Sciences, Malet Street, London WC1E 7HX, UK

## Abstract

We have previously used simple empirical equations to reproduce the literature values of the ionization energies of isoelectronic sequences of up to four electrons which gave very good agreement. We reproduce here a kinetic energy expression with corrections for relativity and Lamb shift effects which give excellent agreement with the literature values. These equations become more complex as the number of electrons in the system increases. Alternative simple quadratic expressions for calculating ionization energies of multielectron ions are discussed. A set of coefficients when substituted into a simple expression produces very good agreement with the literature values. Our work shows that Slater's rules are not appropriate for predicting trends or screening constants. This work provides very strong evidence that ionization energies are not functions of complete squares, and when calculating ionization energies electron transition/relaxation has to be taken into account. We demonstrate clearly that for particular isoelectronic sequences, the ionizing electrons may occupy different orbitals and in such cases more than one set of constants are needed to calculate the ionization energies.

## 1. Introduction

We have previously proposed a simple empirical equation to reproduce the literature values of the ionization energies of one-electron [1] and two-electron [2] atomic ions with very good agreement. This was recently extended to calculate ionization energies and first electron affinities of three and four electron ions [3] which also gave very good agreement with the literature values. However, we used a potential energy approach in our equation which has no theoretical basis.

With the development of quantum theory, the two-particle problem can be solved exactly, and the kinetic energy of the electron in a hydrogen atom or hydrogen-like ion can be calculated using the Schrödinger equation. In 1930, Dirac [4] produced an equation which included a relativistic correction for the electron energy levels. Lamb and Retherford [5–7] were able to show that there is a small shift (now known as the Lamb shift) in the energy levels of the hydrogen atom not included in the Dirac equation. When the Lamb shift is taken into account, the actual ionization energies of one electron atoms are slightly less than that calculated by the Dirac equation. The calculation of the Lamb shift now forms an essential part in the theoretical calculations of energy levels and ionization energies of one and two electron atomic ions [8, 9].

In this work, we begin by showing the expressions with relativistic corrections to calculate ionization energies which give excellent agreement with the literature values. However, as the number of electrons in the sequence increases, the expressions become more and more complex and less predictable. Hence, we discuss alternative simple equations to calculate energies of isoelectronic sequences in excess of five electrons and show that it is more practical to adopt a simple expression to reproduce ionization energies which still provide very good agreement with generally accepted values. To maintain our aim of simplicity, the expressions/equations only contain fundamental constants or values derived from fundamental constants and fairly simple numbers.

## 2. Sources of Data

Moore [10–13] provided very detailed tables of atomic energy levels and ionization potentials in wave numbers (cm^−1^) and values converted from wave numbers to electron volts (eV) (where 1 cm^−1^ equals 1.2398418 × 10^−4^ eV) for atoms and atomic ions with estimated experimental errors and references to original work. These remain the most extensive survey of ionization energies and are still quoted in recent publications. The *CRC Handbook of Chemistry and Physics* [14] contains comprehensive data from Moore and later sources but does not supply information on estimated uncertainties. The majority of published ionization energy data are now available on the *National Institute of Standards and Technology* web site (http://www.nist.gov/srd/). These compilations include values of ionization energies that are accurately measured as well as crude estimates.

## 3. Ionization Energies of the Hydrogen to Boron Isoelectronic Sequences

When an electron in a multielectron system is ionized, we assume that the ionization energy contains (*J*_*k*_) the kinetic energy term; (*J*_*l*_) the Lamb shift term, (*J*_*t*_) the relaxation/transition energy term, and any residual interaction (*E*_*r*_) [2]. Therefore, the ionization energy can be represented as [15]

(1) 1n2μ(Jk−Jl−Jt)+Er=Ek−El−Et+Er,

where *n* is the principal quantum number and *μ* is the reduced mass and are provided in Table 2. *E*_*k*_, *E*_*l*_, and *E*_*t*_ represent *J*_*k*_, *J*_*l*_, and *J*_*t*_ multiplied by 1/*n* ^2^ *μ*, respectively. *J*_*k*_, *J*_*l*_, and *J*_*t*_ are multiplied by 1/*n* ^2^ *μ* because the electron ionized occurs in energy level *n* and it revolves around the centre of mass. A list of reduced masses are given in Table 2. It is common to present ionization energies in eV (electron volt). Calculated results in this work are converted to eV from Joules by using the relationship of 1 eV = 1.60217648 ×  10^−19^ J, any other figures in cm^−1^ are converted to eV by multiplying them with the value 0.00012398418.

## 4. The Relativistic, Lamb Shift, Electron Transition/Relaxation, and Residual Corrections

The energy of an electron moving in a Bohr orbit can be represented by

(2) movo2a0=q1q2(4πεoa02),

where *m*_*o*_ is the electron rest mass, *v*_*o*_ is the velocity of the electron, *q*_1_ *q*_2_ stand for the charges of the electron and nucleus, *ε*_*o*_ is the permittivity of a vacuum, and *a*_0_ is the Bohr radius. The velocity *v*_*o*_ of the electron in the hydrogen atom can be calculated from the above relationship and is equal to 2.1876913 × 10^6^ m/sec. The velocity *v* of the electron in successive atoms of the one-electron series increases by *Z* times where *Z* is the atomic/proton number or *v* = *v*_*o*_ *Z*. When there is more than one electron in the system, we assume that the velocity of the electron changes by (*Z* − *S*) where *S* is the screening constant for that electron.

The theory of relativity [16] points out that the mass *m* of a moving particle is given by the expression $m=m_{o}/(\sqrt{\left(1-v^{2}/c^{2}\right)})$ where *m*_*o*_ is the rest mass of particle. Expansion of this expression gives

(3) m=mo(1+12v2c2+38v4c4+516v6c6+⋯),

therefore it follows that (1/2)*mv* ^2^ = (1/2)*m*_*o*_ *v* ^2^(1 + (1/2)*v* ^2^/*c* ^2^ + (3/8)*v* ^4^/*c* ^4^ + (5/16)*v* ^6^/*c* ^6^ + ⋯).

The kinetic energy components including relativistic correction for a one-electron atom at an equilibrium position (when the relativistic correction is half that of the maximum) are then

(4) 12mov2+0.5(14mov4c2+316mov6c4).

The ionization energy of a one-electron atom is then

(5) I=μ(12mov2+  0.5(14mov4c2+316mov6c4)−El).

The Lamb shift is usually computed by highly complex formulas which require lengthy computer routines to compute [17]. We assume (without theoretical justification) that the Lamb shift is a relativistic charge, mass, and size ratio effect. The reduced mass calculation [18] implicitly assumes that the electron and nucleus are point charges. But they have a finite size hence there needs to be an extra component in the reduced mass calculation. The energy reduction to take account of reduced mass is not simply *m*_*e*_/(*m*_*e*_ + *m*_*p*_)((1/2)*m*_*o*_ *v*_*o*_^2^) but should include a function of the charge and the ratio of nuclear to atomic size, and this component is calculated by the following expression:

(6) El  =(  meme+mp)(α2.67)(12movo2)(Z−S)3.2A1/3,

where *m*_*e*_ is the electron mass, *m*_*p*_ is the proton mass, and the factor for the reduced mass correction for hydrogen is *m*_*e*_/(*m*_*e*_ + *m*_*p*_). The size of the nucleus increases roughly proportional to *A* ^1/3^ [19]. *α* is the fine structure constant, and (*α*/2^.67^) is a crude approximation of the square root of the ratio of nuclear to atomic size for hydrogen, and *A* is the mass number of the atom. In a one electron system, *S* is zero.

After an electron is ionized, one or more of the remaining electron(s) is/are attracted more closely to the nucleus. The attractive energy between the proton and the remaining electron(s) changes because of the change in screening experienced by the remaining electron(s) before and after ionization, [15] and this transition/relaxation energy is a function of

(7) n24(12mo(vo(Z−S1))2−12mo(vo(Z−S2))2),

where *S*_1_ is the screening constant for the remaining electron(s) after ionization and *S*_2_ is the screening constant for the remaining electron(s) before ionization.

In the helium system, there are two electrons and both occupy the 1s orbital. Since each electron occupies half of the space and each is repelled by only one other electron (here we have assumed that the two electrons act as in a two-particle problem and are equivalent), the screening constant is a half (0.5). After ionization, there is zero screening.

In the lithium series, the electron that is ionized occupies a higher (2s) orbital and is shielded by two 1s electrons, and the screening increases by 1 to 1.5. The extra screening experienced by the two inner electrons in the 1s orbital increases to 0.625 and which is an increase by 1/8 or 0.125 rather than 0.5 because the third electron occupies a different orbital and in a different electron shell (i.e., 0.5 of 0.5 of 0.5). After ionization, only two electrons are left in the system and the screening reduces to just 0.5.

In the beryllium series, the electron that is ionized occupies the 2s orbital. Since there are four electrons and each moving in an elliptical orbit, each may at any time interact differently with the other three electrons. The screening of the fourth electron increases by a half to 2, and the other electron in the 2s orbital experiences a screening of between 1.5 and 2 and is 1.75. After ionization of the fourth electron, the screening experienced by the third electron drops back to 1.5.

For the boron system, the outermost electron occupies a new orbital and the screening increases by 1 to 3. The screening of the fourth electron increases by 0.25 to 2.25, and after ionization the screening reduces back to 2.

We assume that there are two opposite and competing residual electron-electron interactions, and this is discussed in detail in previous work and is not fully reproduced here [15]. In summary, there are temporary asymmetric distributions of electrons (e.g., they may be nearer to each other or further apart than average). The first type (when they are nearer to each other) is residual electron-electron repulsion which reduces slightly the energy required to ionize the electron, and this reduction diminishes very rapidly because with each successive ionization the size of the electron orbit/shell becomes smaller and the electrons become much more tightly bound to the nucleus. The reduction in energy resulting from this interaction is expressed by

(8) Er1=(12movo2)  QIα((Z−(I−1))!).

The opposite and competing electron-electron interaction occurs when there are more than two electrons in the system. In a three electron system, two electrons occupy the 1s orbital and one occupies the 2s orbital. The instantaneous asymmetric distribution of electrons produces an effect which result in the 2s electron being screened slightly less than 0.5 from each of the 1s electrons. The result is that one of the electrons is temporarily attracted to the nucleus a bit more than expected and hence increases the amount of energy required to remove it from the atom/ion. This is represented by

(9) Er2=1n2(12movo2(Z−S)2)αQII2,

and the total residual electron interaction energy change is

(10) Er=(−(12movo2)QIα((Z−(I−1))!)   +1n2(12movo2(Z−S)2)αQII2).

Symbols and values of constants shown in all the expressions in this work are given in Table 1. *Q*_*I*_ is the number of electron-electron interactions before ionization and *Q*_*II*_ is the number of electron-electron interactions after ionization.

## 5. Ionization Energies of One and Two Electron Ions

There is only one electron and *n* is 1, the formula for calculating the ionization energy is

(11) I1=μ(12mov2+0.5(14mov4c2+316mov6c4)          −(  meme+mp)(α2.67)(12movo2)(Z)3.2A1/3).

The one-electron ionization energies calculated by (11) when compared with the ionization energies published in the *CRC Handbook of Chemistry and Physics* agree to 99.999% or better in the majority of cases. The biggest absolute difference is 0.086 eV (to 3 decimal places) from a calculated value of 5469.95 eV or 0.00164% [15].

The ionization energy of a two electron system is (*E*_*k*_ − *E*_*l*_ − *E*_*t*_ + *E*_*r*_), where

(12) El=μ((meme+mp)(α22/3)(12movo2)(Z−0.5)3.2A1/3);Et=0.25μ(12mo(vo(Z))2−12mo(vo(Z−0.5))2);Er=(−(12movo2)α((Z)!)).

Since, for simplicity, we have not applied a relativistic correction to *E*_*t*_ we have made a crude approximation of reducing the relativistic correction in *E*_*k*_ by another 5% to 0.45 (rather than 0.5) and where *v* is (*v*_*o*_(*Z* − 0.5)), so it is

(13) Ek=μ(12mov2+0.45(14mov4c2+316mov6c4)).

When compared to the *CRC Handbook of Chemistry and Physics* the majority of values differ by less than 0.01%, the largest absolute difference is 0.216 eV from a calculated value of 2437.846 eV or 0.0089%.

## 6. Ionization Energies of Three-, Four-, and Five-Electron Ions

The ionization energy of a three-, four-, and five electron system is

(14) (Ek−El−Et+Er),

where

(15) El=0.25μ((meme+mp)(α22/3)(12movo2)(Z−0.5)3.2A1/3);Et=μ(12mo(vo(Z−S1))2−12mo(vo(Z−S2))2);Er=((−(12movo2)QIα((Z−(I−1))!)  +1n2(12movo2(Z−S)2)αQII2)).

As with the two-electron system, we have not applied a relativistic correction to *E*_*t*_ but we have made a crude approximation of reducing the relativistic correction in *E*_*k*_ by 5% to 0.45 and where *v* is (*v*_*o*_(*Z* − *S*)), so that the expression becomes

(16) Ek=0.25μ(12mov2+0.45(14mov4c2+316mov6c4)).

The agreement with the literature values of ionization energies is 99% or better in all cases. It is evident from the above discussion that as the number of electrons in the system increases the equations get more complicated and it is difficult to determine the various corrections.

## 7. Alternative Simple Equations to Calculate Ionization Energies

Besides the more complicated equations shown above, ionization energies can be calculated with simpler expressions but not so precise. A simple formula is sometimes used to calculate ionization energies. It often takes the form of [20]

(17) I=(1n∗2)hcRH(Z−S)2,

where *R*_*H*_ is the Rydberg constant for hydrogen (*R*_*∞*_, the Rydberg constant for infinite mass is equivalent to 13.6059 eV), n* is an “effective” quantum number, *Z* is the atomic number, and *S* is the screening constant based on Slater's rules [21] which enable approximations of analytic wave functions to be constructed for rough estimates [22]. Equation (17) makes a very simplistic assumption that when an electron is removed from an atom or ion, the atom/ion remains unchanged except for the removal of that electron. We believe that it is incorrect to use equations like (17) (where the ionization energy is considered as a function of a complete square) to calculate energies in isoelectronic series. For multielectron systems, we need to consider electron transition/relaxation and other smaller components which may influence the ionization energy of an electron.

In a multielectron system, the main components of the energy change during ionization are the electron-proton energy or (*Z*−*S*)^2^ and the electron relaxation energy (or *kn* ^2^[((*Z*−*S*_1_)^2^ − (*Z*−*S*_2_)^2^)] where *k* is a constant dependent on the particular shell/orbital, *S*_1_ and *S*_2_ represent the screening constants of the remaining electrons after and before the electron is ionized). In most cases, the electron-proton energy component alone accounts for 90% or more of the energy change and together with the relaxation energy can represent more than 95% of the total energy change. If factors which in total contribute only a small percentage of the energy change, such as residual repulsion, pairing or exchange energies are excluded, the expression for calculating the ionization energy can be approximated to

(18) I=(1n2)Rμhc ×{(Z−S)2−0.25n2[((Z−S1)2−(Z−S2)2)]}.

This can be expanded to become

(19) I=(1n2)Rμhc{(Z2−2ZS+S2) −k[(Z2−2ZS1+S12)    −(Z2−2ZS2+S22)]}.

Since in the second half of expression (19) the *Z* ^2^ term cancels out, only 2*Z*(*S*_1_ − *S*_2_) and (*S*_2_^2^ − *S*_1_^2^) are left. 2*Z*(*S*_1_ − *S*_2_) can be reduced to *aZ*, and (*S*_2_^2^ − *S*_1_^2^) becomes a constant *b*. This can be rearranged and simplified to

(20) I=(1n2)Rμhc{(Z2−2ZS+S2)−aZ+b},

where *a* and *b* are constants for each isoelectronic system, *n* is the principal quantum number, and, unlike (17), expression (20) is not a complete square.

We have formulated the following rules for working out screening (shielding) constants: (1) for each additional electron in the system, the screening increases by 0.5 unless (2) the electron to be ionized occupies a new orbital such as from beryllium to boron when it increases by 1 and (3) to account for the pairing effect, such as from nitrogen to oxygen, the screening constant increases by 1. For example, for the carbon system, the screening increases by 0.5 units to 3.5 and increases by a further 0.5 to 4 for nitrogen but increases to 5 for oxygen.

We made different estimates of the screening constants *S*_2_ and *S*_1_ and obtained various values of *a* and *b*. We then selected the values that give the best results and a list of screening constants, coefficients *a* and *b* and reduced masses as shown in Table 2. They are used with equation (20) to calculated the ionization energies of isoelectronic sequences from five electrons.

The values calculated from expression (20) are presented for the first six appropriate members of each series for the following two reasons. Firstly, as we have already shown [14], ionization energies of the first few members of isoelectronic sequences are the most precise. Sometimes, only the first four or five members of a series are experimentally measured and uncertainties increase further along a series. Secondly, our results are compared with ionization energies with those compiled in the *CRC Handbook of Chemistry and Physics*, and for many isoelectronic series with more than twenty electrons, only a limited number of values are available for each sequence. Some of these values are given to many significant figures and some only to two or three significant figures because uncertainties can be of the order of 1 eV or higher. Since, as with previous work, all our results are rounded to three decimal places we have decided that where the *CRC Handbook of Chemistry and Physics *has provided values with more than three decimal places they are rounded to three decimal places in the tables.

## 8. Ionization Energies from Five- to Eighteen-Electron Isoelectronic Atomic Ions

Ionization energies reported in the* CRC Handbook* for five to eighteen electronic series (first six members) are given in Table 3. Values of ionization energies calculated using our coefficients are provided in Table 4. Percentage differences between our values and values published by the *CRC Handbook* as listed in Table 5 show that all values agree to 98% or better. Just over 76%, the calculated values agree to 99% or better.

## 9. Ionization Energies from Nineteen- to Twenty-Seven-Electron Isoelectronic Sequences

Treatment of ionization energies of isoelectronic with nineteen electrons or more are different and more complicated. This is because, from atomic number nineteen, the electron to be ionized is in period 4 of the periodic table and such an isoelectronic system includes ionization of s or d electrons [23]. We have demonstrated that for the transition metals or lanthanide [24] elements, the ionization process is complicated and, in the majority of cases, d or f electrons are not removed in the first or second ionization. For example, consider the potassium, calcium, scandium, and titanium series (isoelectronic series with 19, 20, 21, and 22 electrons resp.). For the potassium series (19-electron isoelectronic), the first ionization of potassium and the second ionization of calcium both involve removal of a 4s electron. But the third ionization of scandium and the fourth ionization of titanium (which are in the same isoelectronic series) involve removal of a 3d electron. For the calcium series (20-electron isoelectronic), the first ionization of calcium and the second ionization of scandium involve removal of a 4s electron whereas the third ionization of titanium involves removal of a 3d electron. Similarly with the scandium and titanium series, for the first two members of the series, the energy change is the energy required to ionize a 4s electron but from the third member of the series onwards the energy change is the energy for ionizing a 3d electron (a more detailed discussion is provided in a previous work [23]). Equation (20) shows that ionization is a function of 1/*n* ^2^. Therefore, for the 19, 20, 21, and 22 isoelectronic series, the correct fraction or decimal for 1/*n* ^2^ to use is 1/16 (or 0.0625) for the first two members of the series but 1/9 (or 0.11111) for the remainder of the series. Hence, for the above reasons, it is incorrect to use a single set of coefficients to calculate the energies of an isoelectronic sequence which may involve removal of electrons in different orbitals.

We believe that there is little value calculating the first and second ionization energies of sequences beginning with the potassium. From the potassium series onwards, the third ionization energy requires a different set of coefficients. For example, for the potassium series, a set of coefficients is used to calculate the third ionization energy of potassium, the fourth ionization energy of calcium, and the fifth ionization energy of scandium, and so on because a 3p electron is ionized in all cases. For the scandium series, the third ionization of scandium, the fourth ionization of titanium, and the fifth ionization of vanadium and so on can be calculated using one set of coefficients since in all cases a 3d electron is ionized. However, the third ionization energy of the potassium series and the third ionization energy of the calcium series are identical to the fifth ionization of the chlorine and argon series, respectively. Hence, these two series will not be repeated in Tables 6 and 7.

We have calculated the ionization energies up to the cobalt series (which contains five appropriate published values for comparison) because beyond the cobalt series there are fewer and fewer published values available for use in comparison. Ionization energies of isoelectronic series reported in the* CRC Handbook* for sequences from scandium to cobalt (for each series beginning with the third ionization energy) are given in Table 6. Values of ionization energies calculated using our coefficients are provided in Table 7. Percentage differences between our values and values in the *CRC Handbook *as listed in Table 8 show that all calculated values agree to 98%, or better and just under 83% of the values agree to 99% or better.

## 10. Discussion

We have used a simple quadratic expression in this work. We have not considered exchange and orbital energies (20) and have ignored any residual interactions or relativistic corrections, which for multielectron systems are difficult to apply. Hence, it is not surprising that the agreement with some of the generally accepted values is less than 99%. However, some of the differences between the calculated values and *CRC Handbook* values are less than the experimental uncertainties. However, as we have shown above, equations for solving ionization energies can be very complicated and the results may be unpredictable as the number of electrons in an isoelectronic series increases. Therefore, we believe that there is a strong case to use a simple quadratic expression rather than trying to create complex equations to calculate ionization energies.

Although Slater's rules are still cited in recent publications [25] as adequate for predicting most periodic trends, it has been pointed out that the rules are unreliable when orbitals with a total quantum number of 4 [26] is reached (e.g., a 3p orbital has a principal quantum number of 3, orbital quantum number of 1, and magnetic quantum number of 1, and spin quantum number of 1*⁄*2 already has a total quantum number of 5). Equation (17) and Slater's rules are based on simple assumptions and are unable to account for many different features of ionization energies across the periodic table. We have also shown that ionization energies are not functions of simple complete squares [23], and Slater's rules cannot account for the complex patterns in ionization energies shown in our previous work [24].

## 11. Conclusion

Ionization energies calculated by a kinetic energy approach with simple relativistic and Lamb shift corrections give remarkable agreement with generally accepted values for one- to five-electron isoelectronic series. However, for multielectron isoelectronic series with five or more electrons, there is no accepted methodology for calculating relativistic corrections. Therefore, it is practical and more manageable to use a simple quadratic expression to calculate ionization energies which still give very good agreement with generally accepted values. We have not attempted to calculate ionization energies of sequences beyond cobalt because there are few sequences where a long series of data are available. We believe that expression (20) can be used to calculate ionization energies of any multielectron isoelectronic series assuming that sensible coefficients are applied. It is evident that an equation based on (17) (or function of a complete square such as (*Z*−*S*)^2^) is not the correct representation of the energy change when an electron is ionized. We have also demonstrated that it is incorrect to use a single set of constants/coefficients to calculate the ionization energies of some isoelectronic series such as the potassium, calcium, or scandium series.

Since a great many of the experimental measurements of ionization energies were done over half a century ago, and some of the published values are extrapolated/interpolated, or estimates with fairly large uncertainties there is also a strong case for new measurements to be undertaken and reliability of some of the currently accepted values to be reexamined.

## Figures and Tables

**Table 1:** Symbols, conversion factors, and constants (to 9 significant figures).

Symbol/constant	Value/comment/definition
*α*	0.00729735254
*I*	Number of electrons remaining after ionization
eV	1 electron volt = 1.60217648 × 10^−19^ Joules
*n*	Principal quantum number
*v*	Electron velocity
*Q*	Number of residual electron-electron interactions
*A*	Mass number
*µ*	Reduced mass (see Table 8 for individual values)
*S*	Screening constant for the ionizing electron
*S*_1_	Screening for the remaining electron(s) after ionization
*S*_2_	Screening for the remaining electron(s) before ionization
*Z*	Proton number (nuclear charge)
*c*	Velocity of light 2.99792458 × 10^8^ ms^−1^
*m*_*e*_	Electron rest mass 9.10938215 × 10^−31^ kg
*m*_*p*_	Proton rest mass 1.67262164 × 10^−27^ kg
*q*	Electron charge 1.60217649 × 10^−19^ C
*h*	Planck's constant 6.62606896 × 10^−34^ Js
*ε*_*o*_	8.85418782 × 10^−12^ Fm^−1^

**Table 2:** Coefficients/constants for calculating ionization energies of multielectron ions.

*Z*	(1)	(2)	(3)	(4)
	Screening constants	Coefficient *a*	Coefficient *b*	Reduced mass*
B	3	0.318	0.032	0.999939550
C	3.5	0.572	0.490	0.999954670
N	4	0.842	1.170	0.999961152
O	5	0.716	0.731	0.999966015
F	5.5	1.054	2.357	0.999971388
Ne	6	1.318	3.520	0.999972825
Na	7	2.222	11.809	0.999976377
Mg	7.5	2.344	12.925	0.999977367
Al	8.5	2.882	21.124	0.999979889
Si	9	3.012	22.521	0.999980614
P	9.5	3.194	24.599	0.999982497
S	10.5	2.850	22.194	0.999983051
Cl	11	2.968	23.026	0.999983571
Ar	11.5	3.306	27.683	0.999986454

**	Screening constants	Coefficient *a*	Coefficient *b*	Reduced mass

K	11	2.968	23.026	0.999986113
Ca	11.5	3.306	27.683	0.999986467
Sc	13.5	3.366	30.828	0.999988489
Ti	14	3.832	38.481	0.999989186
V	14.5	4.542	51.618	0.999989405
Cr	15	5.138	62.763	0.999990000
Mn	15.5	5.658	73.472	0.999990188
Fe	16.5	5.850	82.119	0.999990702
Co	17	4.484	57.749	0.999990708

*Reduced masses are calculated from atomic number 5 to atomic number 26; for ions above atomic number 26, the reduced mass of atomic 26 is used since the differences between reduced masses at high atomic numbers are too small to show any differences in the calculated results.

**From the potassium series, the constants apply to calculating ionization energies from the third ionization energy (i.e., third ionization of potassium, fourth ionization of calcium, etc.).

**Table 3:** Ionization energies (eV) of isoelectronic series from the *CRC Handbook* (5 to 18 electron sequences)—first six members of each series.

*Z*	(2)	(3)	(4)	(5)	(6)	(7)
	First	Second	Third	Fourth	Fifth	Sixth
B	8.298	24.383	47.449	77.414	114.243	157.930
C	11.260	29.601	54.936	87.140	126.210	172.180
N	14.534	35.121	62.708	97.120	138.400	186.760
O	13.618	34.971	63.450	98.910	141.270	190.490
F	17.423	40.963	71.620	109.266	153.825	205.270
Ne	21.565	47.286	80.144	119.992	166.767	220.421
Na	5.139	15.035	28.448	45.142	65.025	88.053
Mg	7.646	18.829	33.493	51.444	72.595	97.030
Al	5.896	16.346	30.203	47.222	67.800	91.009
Si	8.152	19.770	34.790	53.465	75.020	99.400
P	10.487	23.338	39.610	59.810	82.660	108.780
S	10.360	23.814	40.740	60.910	84.500	110.680
Cl	12.968	27.630	45.806	67.270	91.650	119.530
Ar	15.760	31.630	50.913	73.489	99.300	128.130

**Table 4:** Ionization energies (eV) of isoelectronic series calculated using expression 23 and coefficients/constants from Table 2 (5 to 18 electron sequences).

*Z*	(2)	(3)	(4)	(5)	(6)	(7)
	First	Second	Third	Fourth	Fifth	Sixth
B	8.306	24.231	46.959	76.490	112.824	155.960
C	11.252	29.715	54.980	87.049	125.920	171.595
N	14.544	35.490	63.238	97.790	139.144	187.302
O	13.616	34.990	63.167	98.148	139.930	188.517
F	17.419	41.045	71.475	108.706	152.741	203.579
Ne	21.564	47.693	80.626	120.361	166.900	220.241
Na	5.090	15.336	28.606	44.900	64.217	86.558
Mg	7.629	19.203	33.801	51.422	72.066	95.734
Al	5.907	16.668	30.452	47.260	67.091	89.945
Si	8.092	20.168	35.267	53.390	74.536	98.706
P	10.489	23.802	40.138	59.497	81.880	107.287
S	10.347	24.179	41.035	60.915	83.818	109.745
Cl	12.955	28.121	46.310	67.523	91.760	119.020
Ar	15.760	31.926	51.116	73.330	98.567	126.828

**Table 5:** Percentage difference between values shown in Tables 3 and 4.

*Z*	(2)	(3)	(4)	(5)	(6)	(7)
	First	Second	Third	Fourth	Fifth	Sixth
B	−0.1	0.6	1.0	1.2	1.2	1.2
C	0.1	−0.4	−0.1	0.1	0.2	0.3
N	−0.1	−1.0	−0.8	−0.7	−0.5	−0.3
O	0.0	−0.1	0.4	0.8	0.9	1.0
F	0.0	−0.2	0.2	0.5	0.7	0.8
Ne	0.0	−0.9	−0.6	−0.3	−0.1	0.1
Na	1.0	−2.0	−0.6	0.5	1.2	1.7
Mg	0.2	−2.0	−0.9	0.0	0.7	1.3
Al	1.3	−2.0	−0.8	−0.1	1.0	1.2
Si	0.7	−2.0	−1.4	0.1	0.6	0.7
P	0.0	−2.0	−1.3	0.5	0.9	1.4
S	0.1	−1.5	−0.7	0.0	0.8	0.8
Cl	0.1	−1.8	−1.1	−0.4	−0.1	0.4
Ar	0.0	−0.9	−0.4	0.2	0.7	1.0

**Table 6:** Ionization energies (eV) of isoelectronic series from the CRC.

*Z*	(2)	(3)	(4)	(5)	(6)	(7)
	Third	Fourth	Fifth	Sixth	Seventh	Eighth
Sc	24.757	43.267	65.282	90.635	119.203	151.060
Ti	27.492	46.709	69.460	95.600	124.980	157.800
V	29.311	49.160	72.400	99.100	128.900	162.000
Cr	30.960	51.200	75.000	102.000	133.000	166.000
Mn	33.668	54.800	79.500	108.000	139.000	174.000
Fe	30.652	51.300	76.060	103.000	134.000	169.900
Co	33.500	54.900	79.800	108.000	140.900	N/A

**Table 7:** Ionization energies (eV) of isoelectronic series using (20) and constants/coefficients in Table 2*.

*Z*	(2)	(3)	(4)	(5)	(6)	(7)
	Third	Fourth	Fifth	Sixth	Seventh	Eighth
Sc	24.780	43.879	66.002	91.149	119.319	150.513
Ti	27.479	47.386	70.316	96.270	125.247	157.248
V	29.330	49.676	73.044	99.436	128.852	161.291
Cr	30.917	51.873	75.853	102.856	132.882	165.932
Mn	33.670	55.351	80.056	107.785	138.537	172.313
Fe	30.642	52.034	76.448	103.887	134.349	167.834
Co	33.509	55.453	80.421	108.413	139.428	N/A

*Ionization energy of isoelectronic series starting from the third ionization energy of the appropriate series.

**Table 8:** Percentage difference between values shown in Tables 6 and 7.

*Z*	(2)	(3)	(4)	(5)	(6)	(7)
	Third	Fourth	Fifth	Sixth	Seventh	Eighth
Sc	−0.1	−1.4	−1.1	−0.6	−0.1	0.4
Ti	0.0	−1.4	−1.2	−0.7	−0.2	0.3
V	−0.1	−1.0	−0.9	−0.3	0.0	0.4
Cr	0.1	−1.3	−1.1	−0.8	0.1	0.0
Mn	0.0	−1.0	−0.7	0.2	0.3	1.0
Fe	0.0	−1.4	−0.5	−0.9	−0.3	1.2
Co	0.0	−1.0	−0.8	−0.4	1.0	N/A

**Table 9:** Coefficients/constants proposed by Agmon to calculate ionization energies of isoelectronic sequences.

*Z*	(2)	(3)
	n*	*S*
B	1.961	3.36
C	1.954	4.09
N	1.952	4.82
O	1.934	5.83
F	1.930	6.57
Ne	1.930	7.32
Na	2.865	8.78
Mg	2.851	9.46
Al	2.849	10.70
Si	2.835	11.40
P	2.814	12.13
S	2.797	13.10
Cl	2.770	13.88
Ar	2.737	14.68

**Table 10:** Ionization energies (eV) calculated using coefficients in Table 9.

*Z*	(2)	(3)	(4)	(5)	(6)	(7)
	First	Second	Third	Fourth	Fifth	Sixth
B	9.511	24.645	46.851	76.130	112.481	155.904
C	12.992	30.159	54.448	85.860	124.394	170.052
N	16.960	36.089	62.355	95.758	136.299	183.977
O	17.119	36.533	63.217	97.172	138.399	186.896
F	21.556	42.949	71.642	107.637	150.932	201.529
Ne	26.220	49.437	79.956	117.776	162.897	215.319
Na	8.165	17.177	29.502	45.141	64.092	86.358
Mg	10.793	20.965	34.482	51.345	71.554	95.109
Al	8.862	18.244	30.976	47.059	66.492	89.276
Si	11.437	21.927	35.800	53.057	73.698	97.723
P	14.145	25.719	40.727	59.170	81.048	106.360
S	14.618	26.437	41.733	60.505	82.754	108.479
Cl	17.251	30.082	46.457	66.377	89.841	116.850
Ar	20.008	33.876	51.375	72.504	97.263	125.653

**Table 11:** Percentage differences between results shown in Table 10 and values from the *CRC Handbook*.

*Z*	(2)	(3)	(4)	(5)	(6)	(7)
	First	Second	Third	Fourth	Fifth	Sixth
B	−14.6	−1.1	1.3	1.7	1.5	1.3
C	−15.4	−1.9	0.9	1.5	1.4	1.3
N	−16.7	−2.8	0.6	1.4	1.5	1.5
O	−25.7	−4.5	0.4	1.8	2.0	1.9
F	−23.7	−4.8	0.0	1.5	1.9	1.8
Ne	−21.6	−4.5	0.2	1.8	2.3	2.3
Na	−58.9	−14.2	−3.7	0.0	1.4	1.9
Mg	−41.2	−11.3	−2.9	0.8	1.8	1.7
Al	−48.1	−11.6	−2.6	0.3	1.9	1.9
Si	−40.3	−10.9	−2.9	0.8	1.8	1.7
P	−34.9	−10.2	−2.8	1.1	1.9	2.2
S	−41.1	−11.0	−2.4	0.7	2.1	2.0
Cl	−33.0	−8.9	−1.4	1.3	2.0	2.2
Ar	−26.9	−7.1	−0.9	1.3	2.1	1.9

## Appendix

We believe it is useful to compare our results with other calculated results (using an equation which is a complete square) to show that our simple model of electron ionization is more realistic and reliable. Agmon [20] published a list of coefficients for the following equation to calculate ionization energies of isoelectronic series:

(A.1) E=hcRH(Z−S)2n∗2,

where *R*_*H*_ is the Rydberg constant for hydrogen. *Z* is the atomic number, *S* is the screening constant, and *n** is an “effective” quantum number.

The list is shown in Table 9. Ionization energies (for five- to eighteen-electron sequences) calculated by the above equation with the set of coefficients are shown in Table 10. Percentage differences with those in the *CRC Handbook* are shown in Table 11.

We have shown that, in general, ionization energies of the first few members (usually the first two or three) of an isoelectronic sequence are most accurately determined, uncertainties normally increase across the higher members of a series. It is clear from Table 10 that the biggest differences between the calculated and accepted values occur where the accuracy and reliability of the accepted values are the greatest. Difference between the calculated and generally accepted values for some of ionization energies are greater than 10%. Secondly, only 13.1% of the calculated figures agree with the accepted ones to 99% or better (as compared to a much higher percentage of ours) and agreement of less (or worse) than 95% occur with over 29.7% of the calculated figures (whereas all our results agree to 95% or better). A major factor is that values of ionization energies are not functions of complete squares, and the equation used by Agmon does not take account of the electron relaxation/transition energy.
